# Research progress on the relationship between mitochondrial function and heart failure: A bibliometric study from 2002 to 2021

**DOI:** 10.3389/fmolb.2022.1036364

**Published:** 2022-10-18

**Authors:** Xiang Qi, Zhide Zhu, Yuhan Wang, Zhihao Wen, Zhixiong Jiang, Liren Zhang, Yan Pang, Jianqi Lu

**Affiliations:** ^1^ Guangxi University of Chinese Medicine, Nanning, Guangxi, China; ^2^ The First Affiliated Hospital of Guangxi University of Chinese Medicine, Nanning, Guangxi, Chinad

**Keywords:** mitochondrial dysfunction, heart failure, bibliometric analysis, visualization, research trend

## Abstract

Heart failure is one of the major public health problems in the world. In recent years, more and more attention has been paid to the relationship between heart failure and mitochondrial function. In the past 2 decades, a growing number of research papers in this field have been published. This study conducted a bibliometric analysis of the published literature on the relationship between MF and HF in the past 20 years by utilizing Microsoft Excel 2019, Biblio metric analysis platform, WoSCC database, VosViewer and Citespace. The results show that the papers have increased year by year and China and the United States are the leading countries in this field, as well as the countries with the most cooperation and exchanges. University of california system is the research institution with the greatest impacts on research results, and Yip H.K. is the author with more papers. The American Journal of Physiology-heart and Circulatory Physiology is probably the most popular magazine. At present, most of the published articles on mitochondria and HF are cited from internationally influential journals. The research focus includes oxidative stress, metabolic dysfunction, mitochondrial Ca^2+^ homeostasis imbalance, mitochondrial quality control and mitochondrial dysfunction mediated by inflammation in the pathogenesis of HF. Targeted regulating of mitochondria will be the keynote of future research on prevention and treatment of HF.

## 1 Introduction

Heart failure (HF), a complex clinical syndrome and the end-stage manifestation of a variety of cardiovascular diseases caused by cardiac structural or functional abnormalities, is one of the major global public health issues characterized by impaired ventricular pumping or filling capacity (van der Meer et al., 2019). Epidemiological studies show that the 5-year survival rate of patients with HF is only 50%, and there are more than 37.7 million HF cases worldwide in 2016 (Ziaeian and Fonarow, 2016). At present, the morbidity, fatality, disability and readmission rate of HF remain high and the figure is expected to continue to rise in the next few decades, especially in the middle-aged and elderly population, placing a huge financial burden on patients (Yancy et al., 2013; Yancy et al., 2017). A report says that the 30-day all-cause readmission rate for HF in the United States is 19%, the all-cause mortality rate for acute HF in European countries is 17%, and 19.2% of HF patients in Asia died or were readmitted to hospital due to HF within 1 year (Rossignol et al., 2019). The understanding of the pathogenesis of HF has evolved from hemodynamic abnormalities to neuroendocrine abnormalities, and then to ventricular remodeling. However, these mechanisms do not fully explain the complexity of HF pathophysiology. Therefore, it is necessary to clarify the pathogenesis and explore new treatment options to delay or reverse the progress of HF. In recent years, the relationship between HF and mitochondrial function (MF) has been paid more and more attention. Researchers have noticed that mitochondrial dysfunction is widespread in the process of HF (Brown et al., 2017). The heart is a high energy-consuming organ and the most active organ in human metabolism which accounts for only 0.5% of human body weight. However, its ATP consumption accounts for 8% of the total, and about 95% of the ATP needed by the heart comes from the oxidative metabolism of mitochondria. Mitochondrion is a double-membrane organelle that exists in almost all eukaryotic cells. Its main function is to produce ATP by oxidative phosphorylation. The mitochondrial content of the heart is the highest in all human tissues, and myocardial mitochondria must operate efficiently to maintain the normal needs of the human body (Neubauer, 2007; Sabbah, 2020). Mitochondrial dysfunction is very common in the pathogenesis. Studies have found that changes in metabolic substrate utilization, increased oxidative stress, Ca^2+^ homeostasis imbalance, mitochondrial quality control imbalance and inflammatory response can directly or indirectly lead to mitochondrial dysfunction, and ultimately lead to irreversible damage of cardiac function (O'Rourke et al., 2021; Del et al., 2021; Dubois-Deruy et al., 2020; Keshavarz-Bahaghighat et al., 2020). Therefore, ensuring the normal function of myocardial mitochondria and maintaining myocardial energy metabolism have become new ideas for the treatment of HF. In the past 2 decades, the research on the relationship between HF and MF has been paid more attention, and the number of literature published has been increasing, but there is a lack of research in this field from the perspective of bibliometrics.

Introduced by Pritchard in 1969, bibliometrics is a discipline that applies mathematical and statistical methods to the study of books and other documents (Ma et al., 2021). This method can quickly extract useful information from a large number of literatures, help scholars grasp the research hotspots and development trends in a certain field instantly, and also evaluate the distribution of countries, institutions, authors and journals in the research field, so as to lay a foundation for future research. In addition, bibliometrics can also be used to evaluate the trend of literature research qualitatively and quantitatively. On the one hand, this method can quickly and accurately present the most influential research results in a certain field based on public databases, providing a theoretical basis for further research. On the other hand, the use of these information can also provide reference for decision makers. In this study, researchers conducted a bibliometric analysis of the literatures published from 2002 to 2022 on the relationship between MF and HF, summarized the development status and future research trends and hotspots in this field according to the analysis results, and provided reference and directions for future research.

## 2 Materials and methods

### 2.1 Data collection

The bibliometric analysis in the paper is based on the Web of Science Core Collection (WoSCC), the quality of the literature included in WoSCC is high and constantly updated, which can effectively ensure the quality of literature analysis (Zhang et al., 2020a; Zhang et al., 2020b). The search time is from 1 January 2002 to 31 December 2021.

**FIGURE 1:**
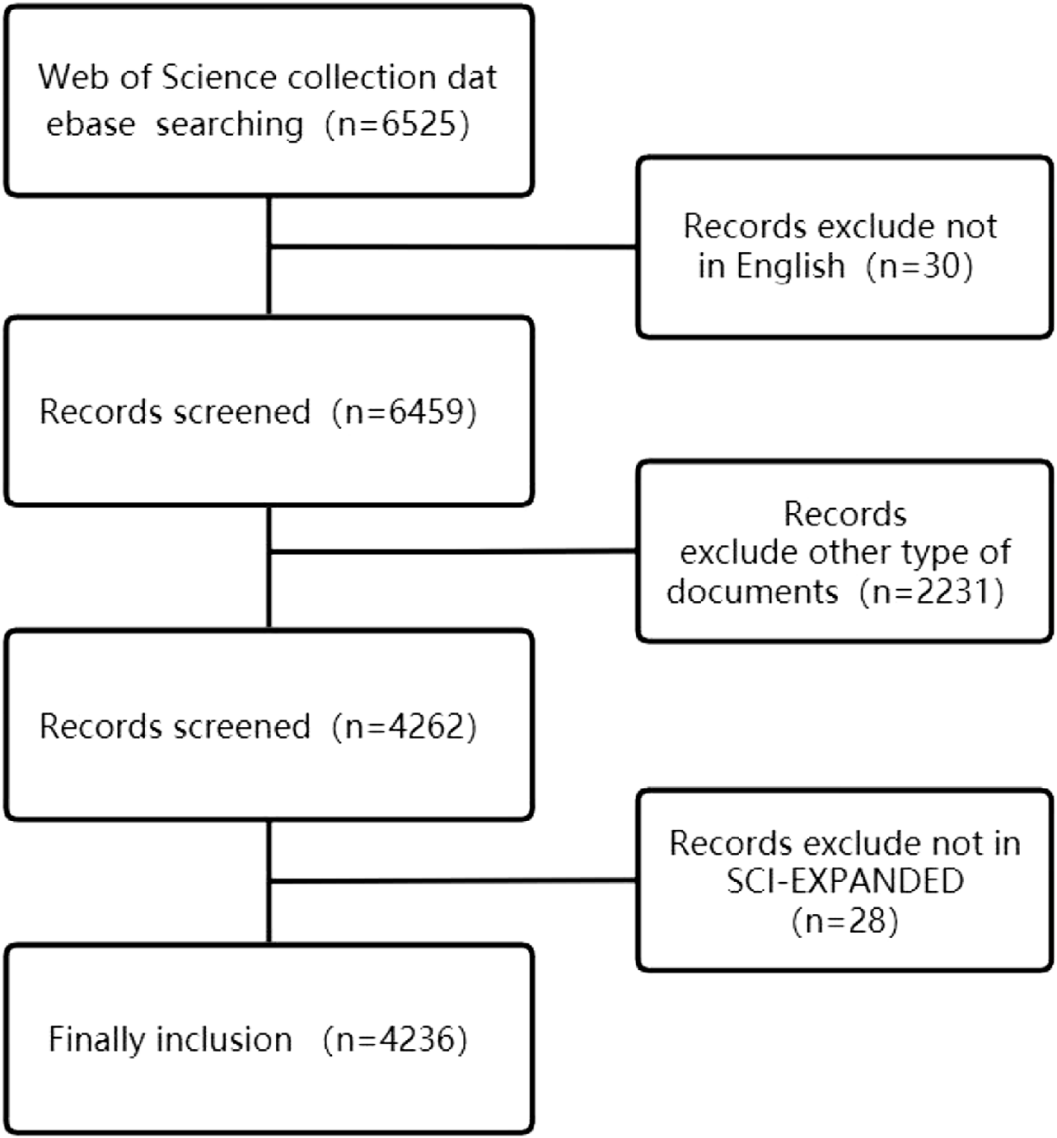
Flowchart of literature selection.

In order to ensure the accuracy of the search, all searches will be completed on 10 March 2022. The search term is {[(TS = (Heart Failure)] OR TS = (Myocardial Failure) OR TS = (Cardiac Failure)} AND {[(TS = Mitochondrion) OR TS = (Mitochondria)] OR TS = (Mitochondrial)}. 6514 articles were retrieved, and the retrieved articles were further screened by two researchers according to the following criteria: 1) excluding non-English articles; 2) excluding the literature whose publication category is not “article”; 3) excluding non-SCI-E indexed literature; After screening, a total of 4236 documents were included, and further documents were exported in document format for analysis. The screening criteria are shown in Figure1.

### 2.2 Data analysis

In this study, the literature retrieved by WoSCC was imported into Microsoft Excel 2019, Bibliometric analysis platform, VosViewer and Citespace for visual analysis. Developed by Professor Chen Chaomei, CiteSpace is a visual analysis software based on JAVA environment and dedicated to the analysis of potential information in scientific literature. It can present the development rules and distribution of scientific knowledge in a certain field by visual means. Based on the existing data, the key points in the development of the field are identified, especially the turning points and key points in the field of knowledge (Chaomei, 2006). VOSviewer is a visualization software developed by the Science and Technology Research Center in Leiden of Netherlands, through which visual network maps can be constructed based on literature information, and ultimately a comprehensive understanding of the scientific structure and dynamic development trend of a field can be achieved (van Eck and Waltman, 2010). Bibliometric online analysis platform (https://bibliometric.com/) can make online bibliometric analysis of scientific citation data and provide valuable scientific information for researchers with simple operation methods and the most intuitive expression. It gives scholars a clear grasp of the discipline structure and relationship, research content and focus in a certain field, and it predicts the Frontier and development trend.

In this study, the countries/regions, institutions, authors, journals, references, keywords, citations, H index (the number of papers with citation number ≥ H) and impact factor (IF) in 2021were analyzed. Microsoft Excel 2019 was used to analyze the annual H index and publishing trends in this field, and knowledge maps were drawn through Citespace, VosViewer and online bibliometric.

## 3 Results

### 3.1 Global publications and trends

**FIGURE 2:**
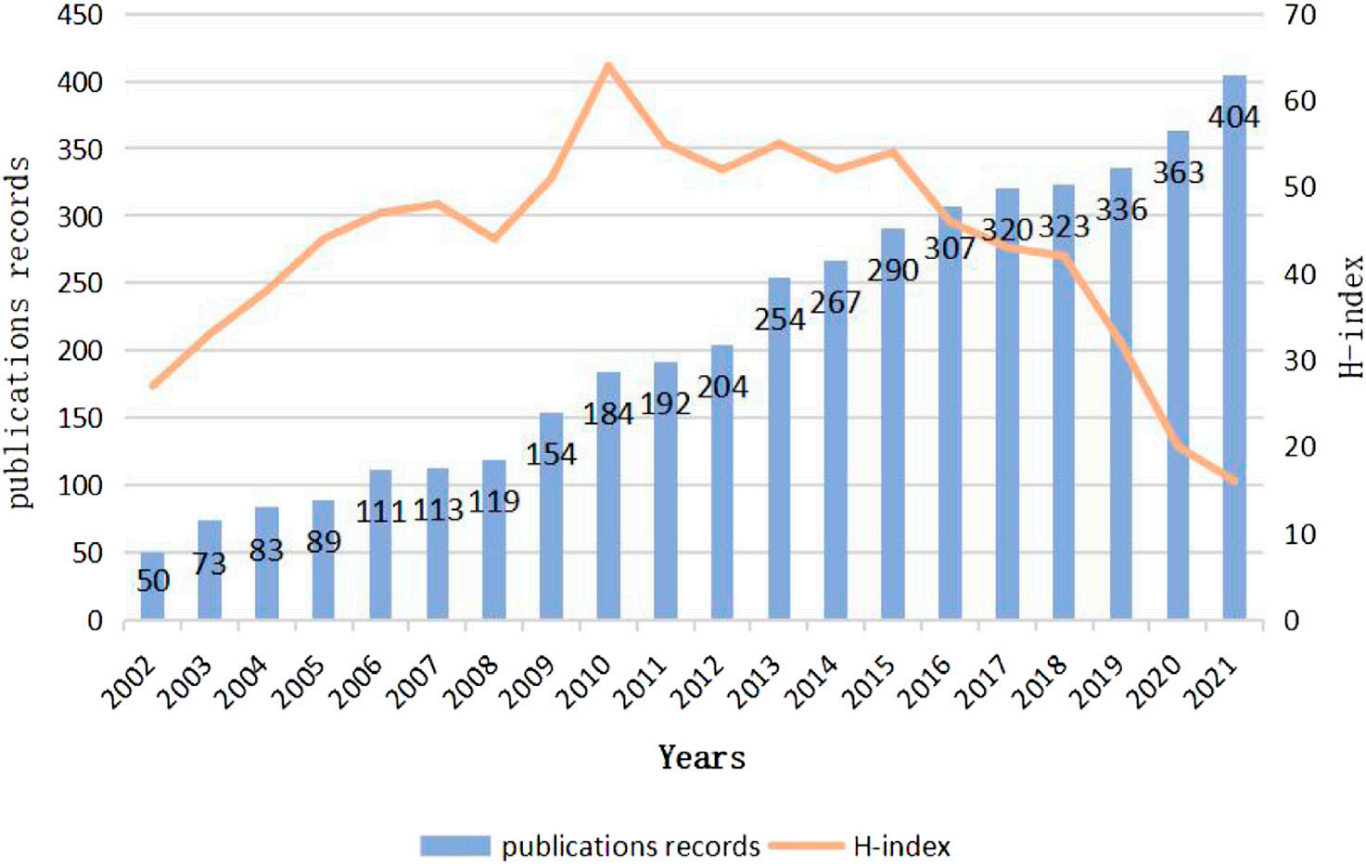
Global number of publications and H-index of publications in the field of relationship between Mitochondrial function and heart failure from 2002 to 2022.

By analyzing the change of the number of publications in a certain field over time, we can effectively evaluate the historical process and current research status and predict the future development trend (Gao et al., 2020). It can be seen from (Figure2)that the number of literatures published on the relationship between MF and HF is increasing year by year from 2002 to 2021. The number of research literatures is small from 2002 to 2008, which indicates that the research on the relationship between HF and MF is still in its infancy during this period. After 2009, articles published increased rapidly until the output reached 404 in 2021. In addition, From the H index distribution, we can see that the year of 2010 is the highest, which shows that the papers published in this year have great influences and far-reaching significance. From the perspective of overall trend, more and more scholars are expected to participate in the future research. It has great potential to study HF from the aspect of mitochondrial dysfunction.

### 3.2 Distribution of countries/regions

**FIGURE 3:**
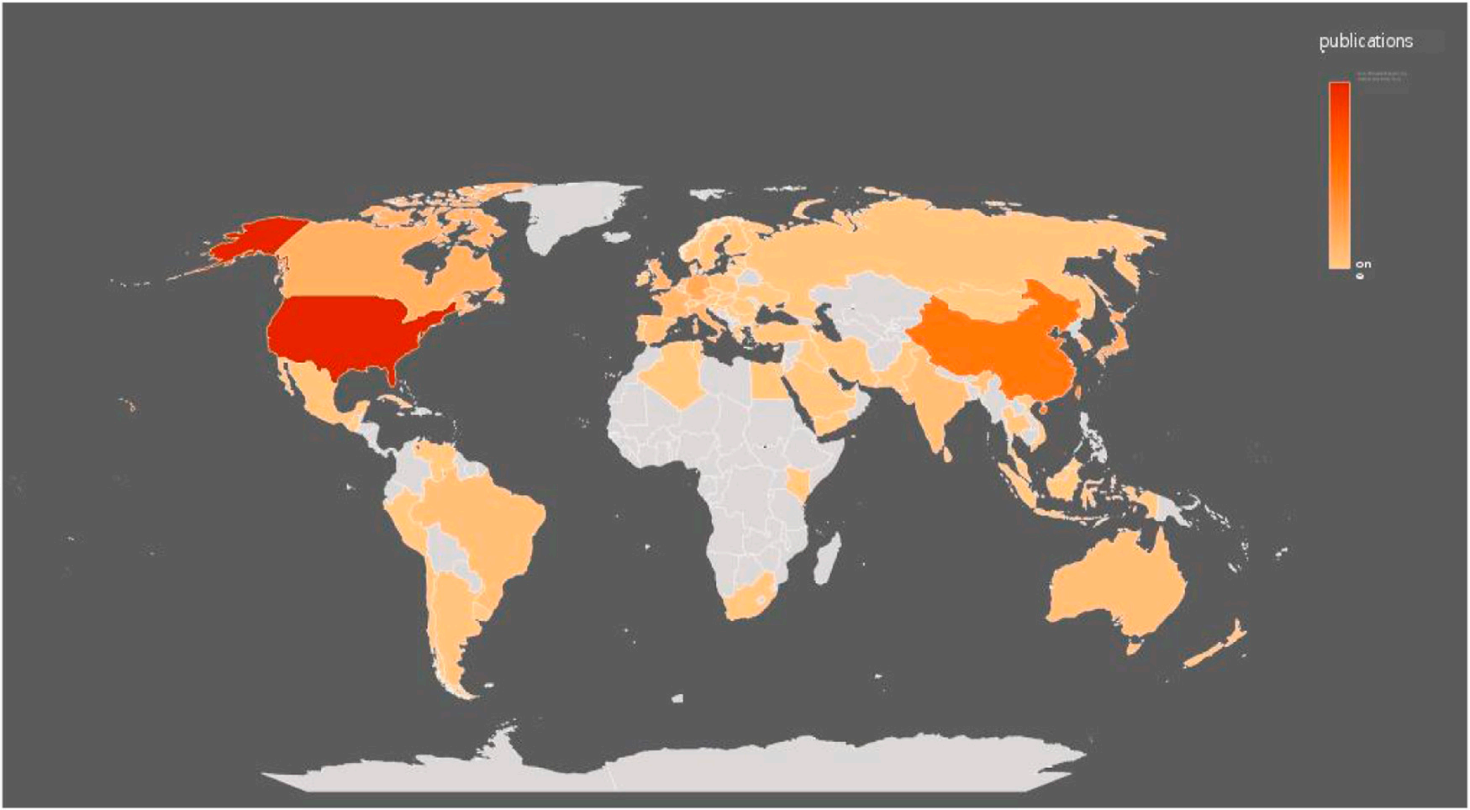
Spatial distribution of global publications.

**FIGURE 4:**
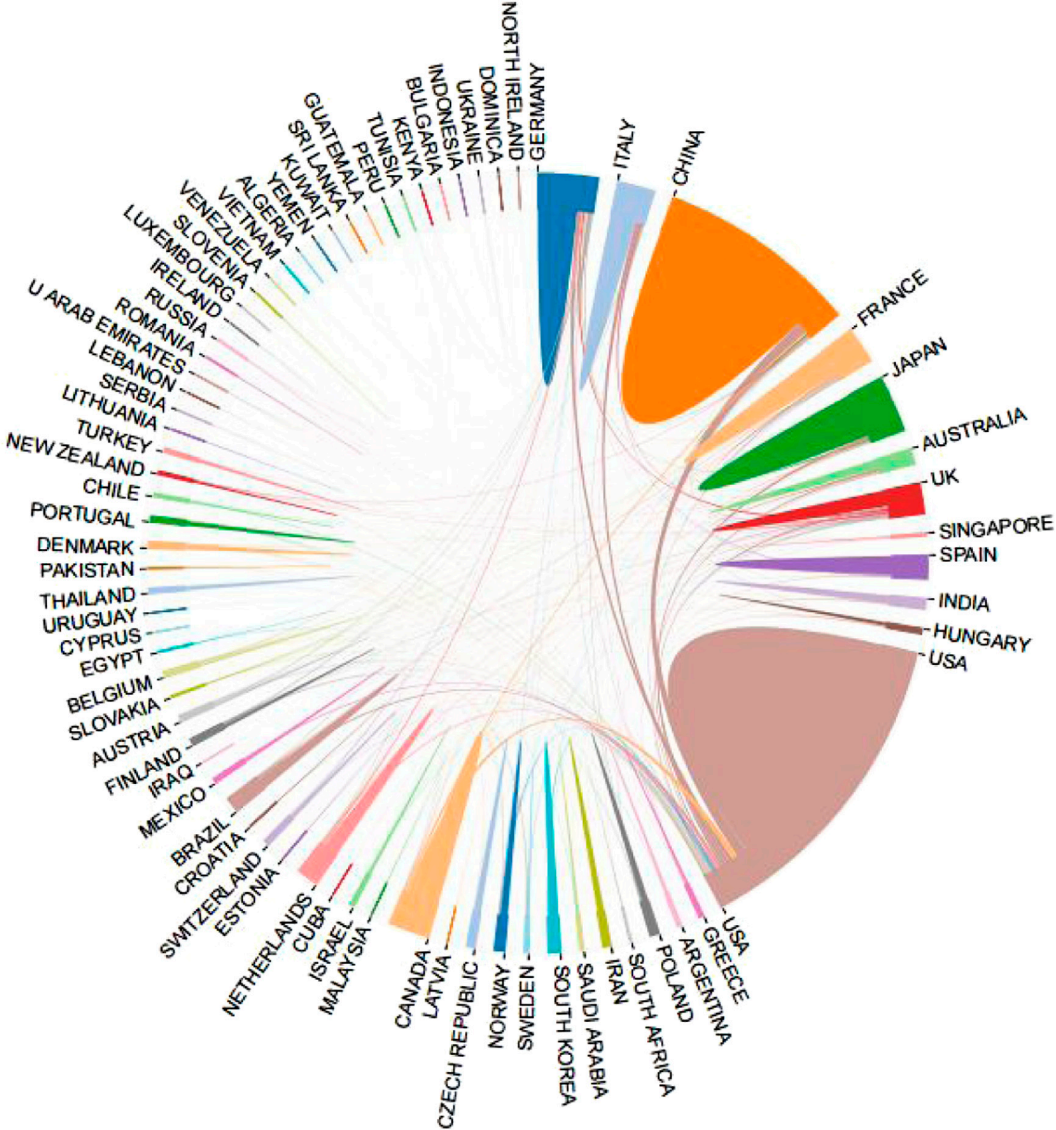
International collaboration between country/region.

After visualized analysis of the publications, it was found that 79 countries/regions participated in the study (Figure 3), of which the United States (1744) and China (877) had the highest number of publications, almost equal to the sum of the other countries/regions. The next goes to Japan (336), Germany (335), Canada (255), United Kingdom (237), Italy (210), France (168), Taiwan (145) and Netherlands (129). In terms of H index, the United States (128) is the highest, followed by Germany (Wai et al., 2015), China (Gao and Hu, 2021), Canada (Lewis and Lewis, 1914) and Japan (Koentges et al., 2015) as more details can be seen in Table 1. In addition, from the perspective of cooperation among countries, the United States, which has the largest number of publications, has active cooperation with other countries, while China is the most cooperative country of the United States (Figure 4).

**TABLE 1:** Top 10 the most productive countries/regions on mitochondrial function and heart failure.

Rank	Country/region	Publications	H-index
1	UNITED STATES	1744	128
2	CHINA	877	63
3	JAPAN	336	56
4	GERMANY	335	65
5	CANADA	255	58
6	UNITED KINGDOM	237	51
7	ITALY	210	44
8	FRANCE	168	46
9	CHINA^,^S TAIWAN	145	28
10	NETHERLANDS	129	35

### 3.3 Distribution of institutions

**FIGURE 5:**
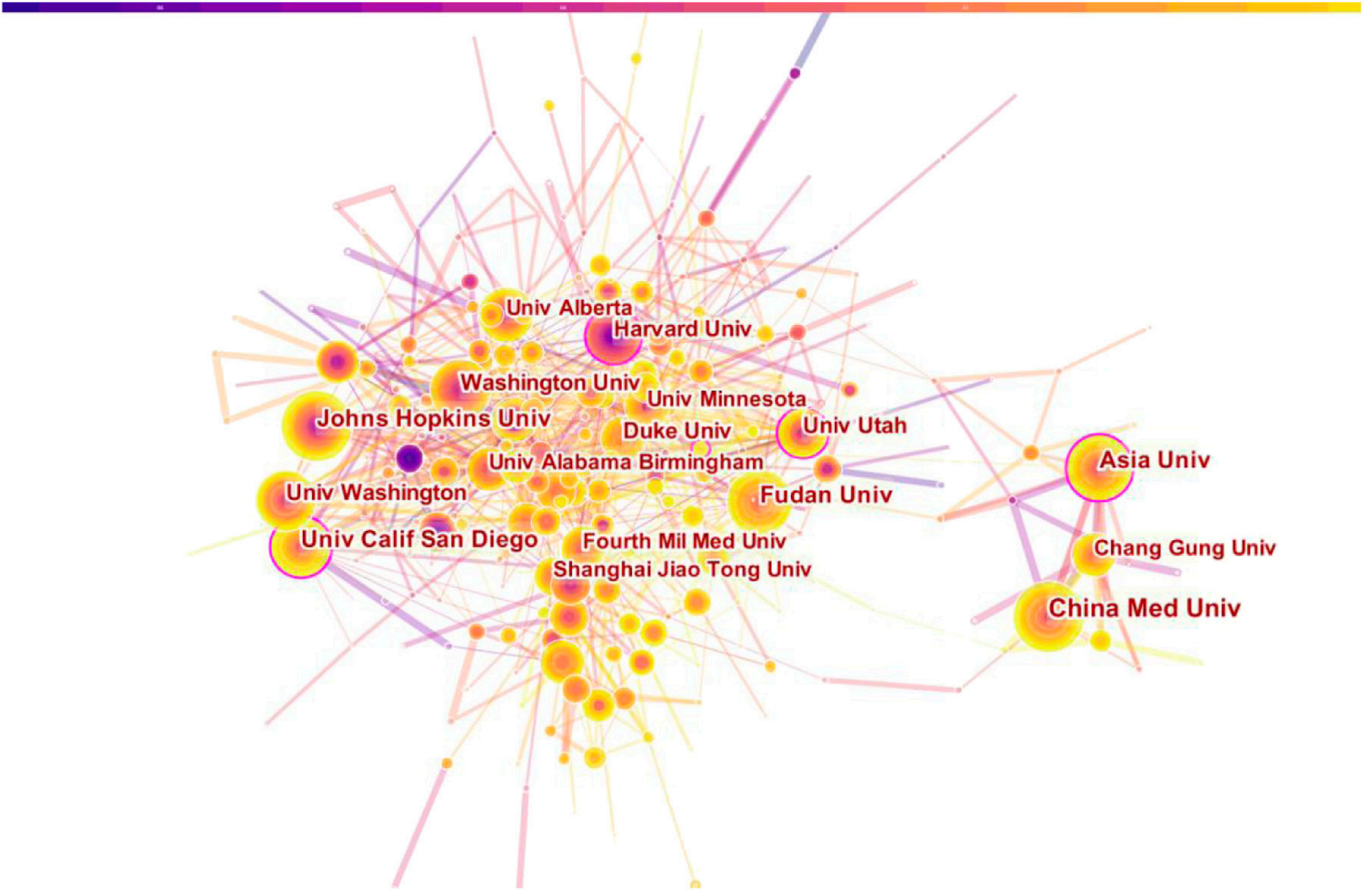
Distribution of publications from different institutions.

VosViewer was used to visually analyze the publishing institutions. The results showed that a total of 3838 institutions were involved in and the top ten organizations of publications and H index were analyzed. UNIVERSITY OF CALIFORNIA SYSTEM 185) has the most papers, then goes to INSTITUT NATIONAL DE LA SANTE ET DE LA RECHERCHE MEDICAL E INSERM (125), UNIVERSITY OF TEXAS SYSTEM (119), and HARVARD UNIVERSITY (112). HARVARD UNIVERSITY 8374) was cited most frequently, followed by UNIVERSITY OF TEXAS SYSTEM 7811) and UNIVERSITY OF CALIFORNIA SYSTEM (7671). From the analysis of H index, the institutions with the highest H index are UNIVERSITY OF TEXAS SYSTEM (Odagiri et al., 2009) and UNIVERSITY OF CALIFORNIA SYSTEM (Odagiri et al., 2009). Therefore, it can be inferred that UNIVERSITY OF TEXAS SYSTEM and UNIVERSITY OF CALIFORNIA SYSTEM have greatest impacts in this field. In terms of cooperation among various agencies, Institutions in the United States have more cooperation with institutions in France, United Kingdom and China, while institutions in Taiwan mainly cooperate with Asia University, Chang Gung University and China Medical University (See Table 2 Figure 5 for specific results). In a word, the above analysis results show that the research institutions in the United States have studied the relationship between MF and HF more deeply, which have more cooperation with other national institutions and greater influence in this field.

**TABLE 2:** Top 10 the most productive institutions.

Rank	Institution	Documents	Ciation	H-index
1	UNIVERSITY OF CALIFORNIA SYSTEM(UNITED STATES)	185	7671	46
2	INSTITUT NATIONAL DE LA SANTE ET DE LA RECHERCHE MEDICALE INSERM(FRANCE)	125	6767	40
3	UNIVERSITY OF TEXAS SYSTEM(UNITED STATES)	119	7811	46
4	HARVARD UNIVERSITY(UNITED STATES)	112	8374	41
5	UNIVERSITY OF TEXAS SYSTEM(UNITED STATES)	92	4012	36
6	VETERANS HEALTH ADMINISTRATION VHA (UNITED STATES)	87	3814	35
7	JOHNS HOPKINS UNIVERSITY(UNITED STATES)	78	3589	32
8	UNIVERSITY OF CALIFORNIA SAN DIEGO(UNITED STATES)	76	3377	30
9	UNIVERSITY OF LONDON(UNITED KINGDOM)	72	3380	27
10	CHINA MEDICAL UNIVERSITY TAIWAN(CHINA)	71	1409	24

### 3.4 Distribution of authors

**FIGURE 6:**
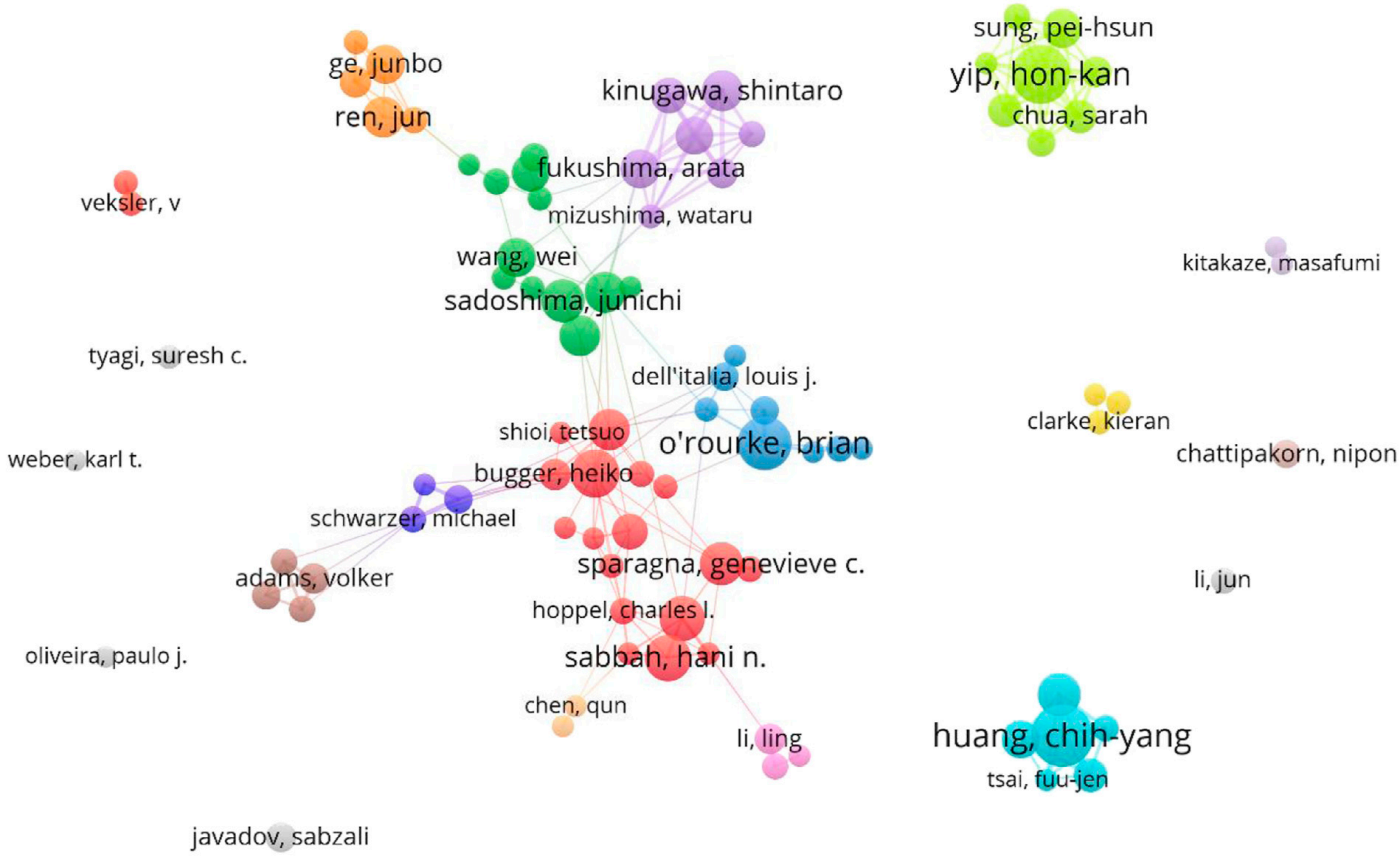
VOSviewer visualization map of authors involved in mitochondrial function and heart failure.

VOSviewer was used to visually analyze the authors’ posts and cooperation, and then publications of the top ten authors were exported to Table 3. The results show that there are 24,919 authors involved in the study. The table shows the number of publications, H index, cooperation of the top ten authors in this field. According to the analysis, Yip H. K., Huang C. Y. and O. ′Rourke B. have published more papers, while Huang C. Y., O.′ Rourke B. and Abel E. D. have higher H index, indicating that they have made some achievements in this field. In addition, according to the analysis of author cooperation (Figure 6), the authors worldwide are scattered and have not yet formed cooperation teams of a certain scale except Wang Wei’s. However, the Yip H. K. team and Huang C. Y. team from Taiwan do not have in-depth cooperation with other countries/regions.

**TABLE 3:** Top 10 authors in the field of mitochondrial function and heart failure.

Rank	Authors	Institution	Documents	H-index
1	Yip H. K.	Chang Gung Univ	30	12
2	Huang C. Y.	China Med Univ	27	19
3	O’rourke B.	Johns Hopkins Univ	24	18
4	Abel E. D.	The University of Utah	19	20
5	Ren jun	University of Wyoming	19	15
6	Sung pei-hsun	Chang Gung Univ	19	9
7	Lopaschuk G. D.	University of Alberta	18	14
8	kinugawa shintaro	Hokkaido Univ	17	12
9	Kuo W. W.	China Med Univ	17	17
10	Wang wei	Shanghai Jiao Tong Univ	17	15

### 3.5 The analysis of Co-cited journals and journals

**FIGURE 7:**
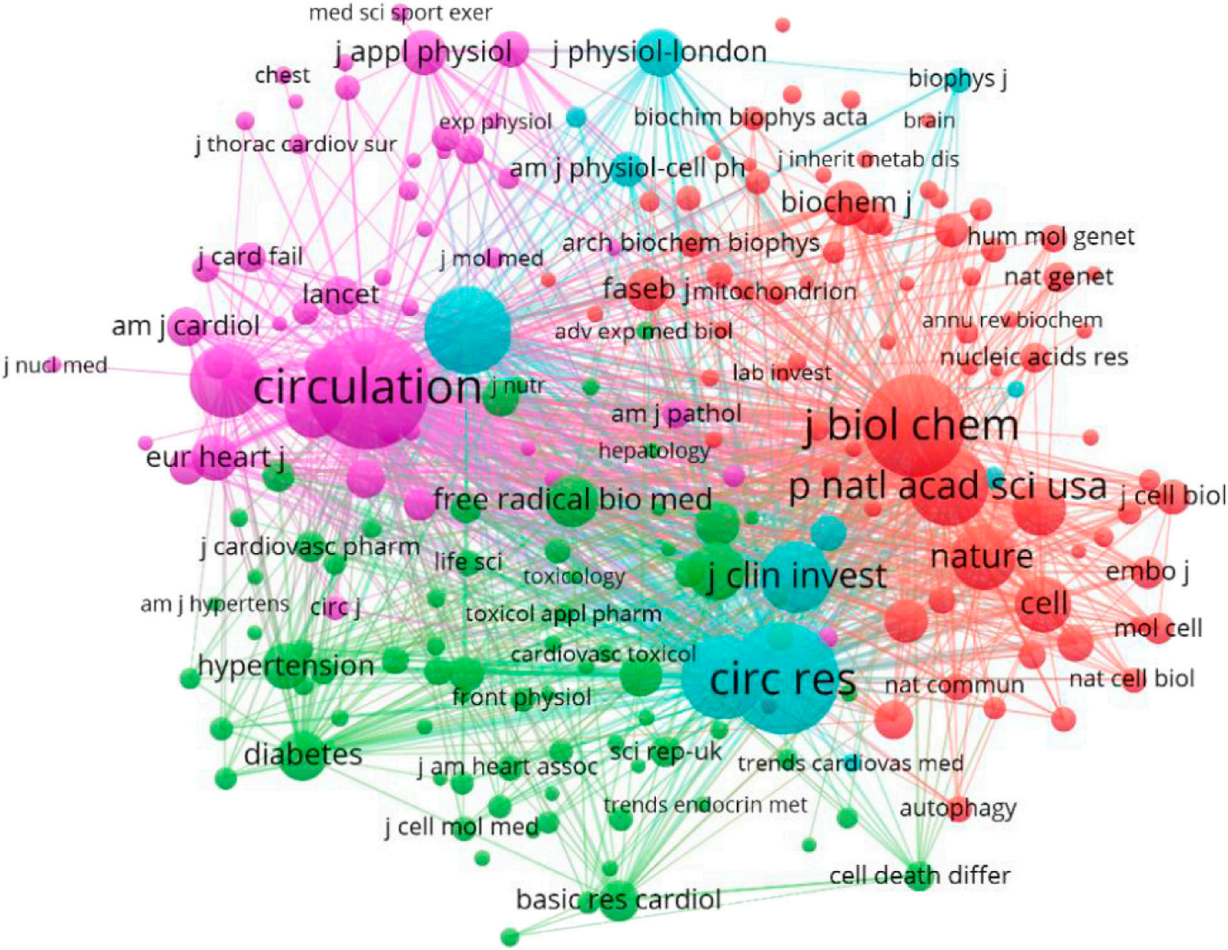
VOSviewer visualization map of co-cited journals.

From 2002 to 2022, 832 journals reported the relationship between HF and MF. In this study, VosViewer was used to analyze the top 10 journals. American Journal of Physiology Heart and Circulatory Physiology (176), The Journal of Molecular and Cellular Cardiology (145) and PLoS One (140) reported the most. Circulation (9150) and Circulation Research (7884) were cited most frequently and had the highest H index. This shows that the literature published in the above two journals has great influence in this field. The impact factors of these journals range from 2.919 to 25.499, among which PLoS One has the lowest IF and Circulation has the highest, the JCR partition, Q1 (30%), Q2 (40%) and Q3 (30%) (Figure 7 and Table 4). Based on the above analysis, it can be inferred that American Journal of Physiology-heart and Circulatory Physiology is probably the most popular journal in this field.

**TABLE 4:** Top 10 journals related to mitochondrial function and heart failure research.

Journal	Documents	Citations	2021 IF	JCR
American journal of physiology-heart and circulatory physiology	176	6665	4.215	Q2
Journal of molecular and cellular cardiology	145	4399	4.729	Q2
Plos one	140	3705	2.919	Q3
Circulation	86	9150	25.499	Q1
Circulation research	84	7884	15.899	Q1
Scientific reports	76	1228	4.130	Q3
Cardiovascular research	71	1226	8.660	Q1
Journal of biological chemistry	51	2969	4.500	Q2
Free radical biology and medicine	48	1643	6.401	Q2
International journal of cardiology	47	1019	3.622	Q3

### 3.6 The analysis of Co-cited references

Co-citation is a research method to measure the degree of relationship between articles. If two or more articles are cited by one or more papers at the same time, the two articles are considered to be co-cited. In this part of the study, the top ten cited articles in the 4236 articles were selected for analysis. According to the analysis results, the above articles were published between 1999 and 2010, and the IF was between 5.182 and 91.245, of which 90% were in the JCR Zone 1, and six articles have been cited more than 100 times (Table 5). Most of the cited literatures focused on exploring the pathogenesis of HF from the perspective of cardiac energy metabolism, mitochondrial quality control and oxidative stress.

**TABLE 5:** Top 10 co-cited references related to mitochondrial function and heart failure.

Rank	References	Citations	Years	2021 if	JCR
1	The failing heart—An engine out of fuel	221	2007	91.245	Q1
2	Myocardial substrate metabolism in the normal and failing heart	167	2005	37.312	Q1
3	Depressed mitochondrial transcription factors and oxidative capacity in rat failing cardiac and skeletal muscles	118	2003	5.182	Q2
4	Oxygen, oxidative stress, hypoxia, and heart failure	115	2005	14.808	Q1
5	Myocardial Fatty Acid Metabolism in Health and Disease	112	2010	37.312	Q1
6	Peroxisome proliferator-activated receptor gamma coactivator-1 promotes cardiac mitochondrial biogenesis	102	2000	14.808	Q1
7	Cardiac Metabolism in Heart Failure Implications Beyond ATP Production	99	2013	17.367	Q1
8	Mitochondrial electron transport complex I is a potential source of oxygen free radicals in the failing myocardium	99	1999	17.367	Q1
9	Mitochondrial energy metabolism in heart failure: a question of balance	96	2005	14.808	Q1
10	Mitochondrial DNA damage and dysfunction associated with oxidative stress in failing hearts after myocardial infarction	94	2001	17.367	Q1

### 3.7 The analysis of keywords

**FIGURE 8:**
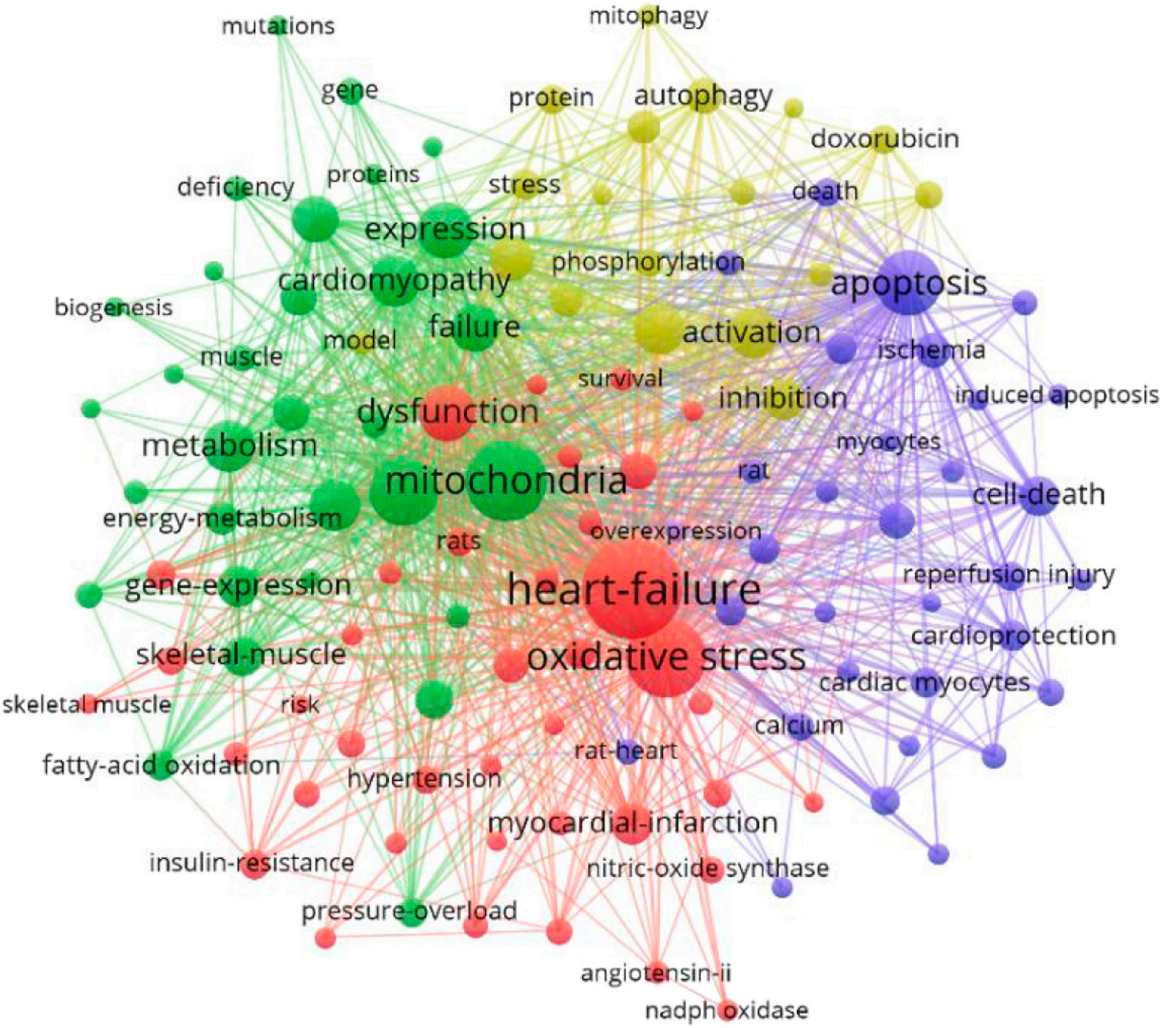
VOSviewer visualization map of keywords clustering analysis.

**FIGURE 9:**
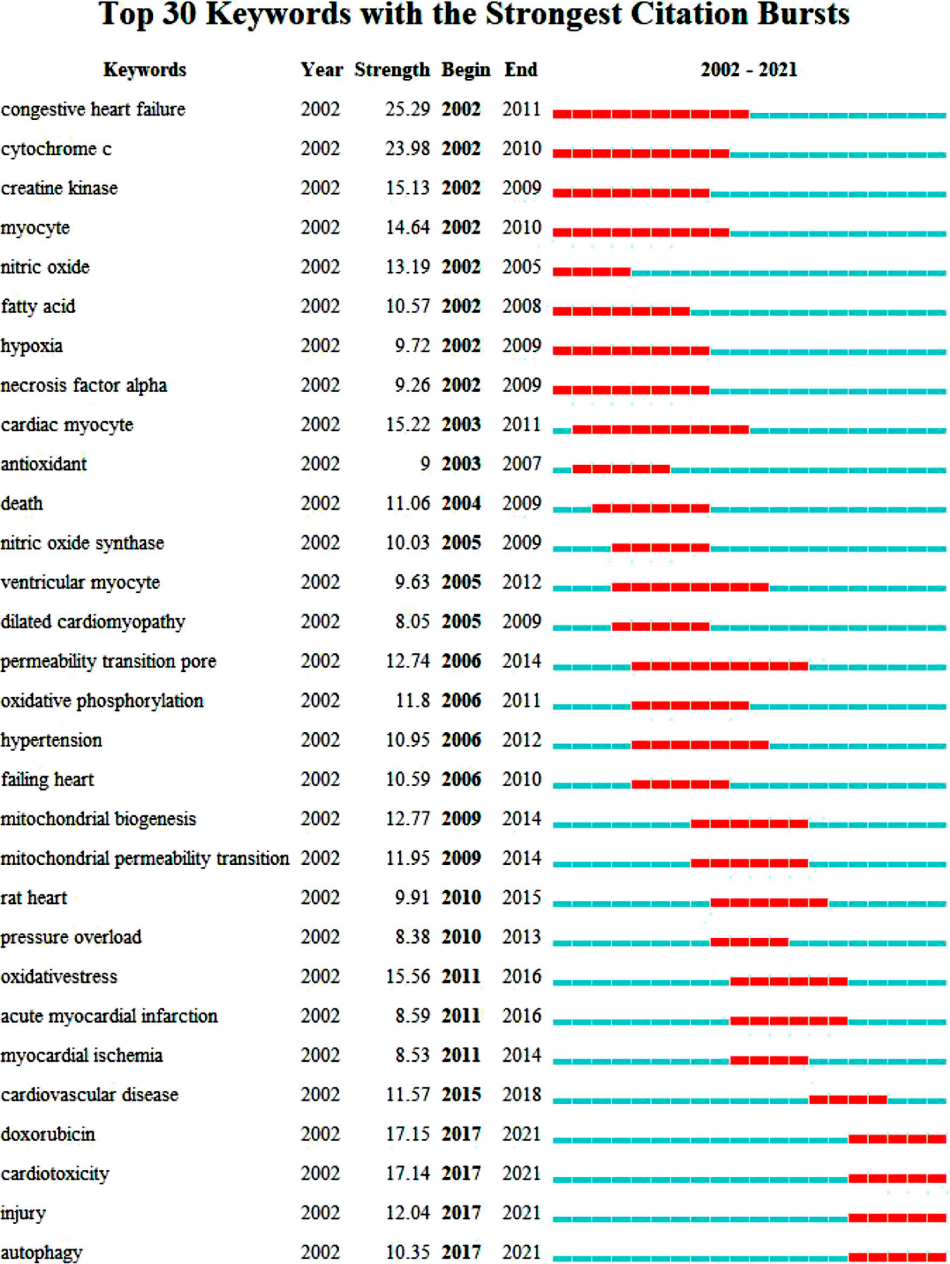
CiteSpace visualization map of top 30 keywords with the strongest citation bursts.

Key words are the core of a document. Through the summary of key words, we can intuitively understand the research hotspots and trends in a field. There are 5958 keywords in the 4236 articles of this study. VOSviewer and CiteSpace were used to visualize and analyze all the keywords to make a keyword co-occurrence map (Figure 8) and a keyword emergence map (Figure 9), and then the top 30 keywords were exported to Table 6. It can be seen from the keyword cluster map made by VOSviewer that it can be divided into four clusters according to different colors, namely red, green, purple and yellow. HF, oxidative stress and myocardial infarction were the main keywords in the red cluster, and mitochondrial, metabolism and myocardial infarction were the main keywords in the green cluster. The main keywords of yellow clustering of cardiomyopathy were activation, inhibition and autophagy, and the main keywords of purple clustering were apoptosis, cell death and calcium. Keyword emergence refers to the rapid increase in the citation frequency of the keyword in a certain period of time. Through this method, we can understand the development process and research hotspots of a research field. CiteSpace was used to analyze the emergence of keywords and further predicted the development trend in the past 20 years. From 2002 to 2010, there were many studies on the relationship between congestive HF and cytochrome C, TNFα and creatine kinae. From 2010 to 2017, scholars paid more attention to the relationship between HF and oxidative stress, mitochondrial dysfunction, mitochondrial permeability transition pore and oxidative phosphorylation. It was seen that key words such as doxorubicin, injury, cardiotoxicity and autophagy have emerged since 2017. It can be inferred that the cardiotoxicity of doxorubicin has been increasingly recognized and used in animal models of HF in recent years (Cappetta et al., 2017). At the same time, the study of mitophagy is also a hot spot in recent years. This study also excluded the high-frequency keywords related to the search topics such as heart-failure and mitochondria, and then interpreted the top keywords. The results showed that in the past 20 years, scholars focused mostly on the relationship between oxidative stress, apoptosis and mitochondrial dysfunction in the pathogenesis of HF and cardiometabolic effects.

**TABLE 6:** Top 30 keywords related to mitochondrial function and heart failure.

Rank	Count	Keywords	Rank	Count	Keywords
1	894	oxidative stress	16	266	Inhibition
2	635	apoptosis	17	239	Energy metabolism
3	479	dysfunction	18	225	Rat
4	466	expression	19	208	Protein
5	414	metabolism	20	203	Mice
6	399	cardiomyopathy	21	194	Autophagy
7	389	activation	22	192	Cell
8	387	hypertrophy	23	191	Nitric oxide
9	373	myocardial infarction	24	189	Dilated cardiomyopathy
10	359	mechanism	25	184	Inflammation
11	311	cardiac hypertrophy	26	182	Ischemia
12	299	skeletal muscle	27	162	Fatty acid oxidation
13	285	cell death	28	157	Mitochondrial function
14	274	gene expression	29	152	Injury
15	270	cardiomyocyte	30	149	*In vivo*

## 4 Discussion

### 4.1 Research results

In this study, we analyzed the development process, research status and hot trends of the relationship between MF and HF in the past 20 years. From the overall trend of the number of papers published in this field, more and more scholars pay attention to the role of MF in the pathogenesis of HF, especially in the past 6 years, the number of papers published has increased greatly. Therefore, it can be inferred that in recent years, people’s understanding of MF has become more mature, and there are more related basic studies and the intervention of HF from MF may become a hot spot and trend in the prevention and treatment of HF in the future.

At present, there are many studies on the relationship between MF and HF in the United States, and extensive cooperation has been reached with other countries/regions, while China and the United States have the most cooperative studies. In addition, researchers in developed countries/regions such as Europe and the United States have paid more attention to the relationship between MF and HF. From the visualized analysis of the institutions, the institutions with more research and publications are universities and research institutes in the United States, and the United States has more cooperation with research institutions in China and other developed European countries, but there are still some countries/regions with less cooperation with foreign countries. Therefore, it is suggested that research institutions around the world should actively cooperate in the future to explore the role of MF in the prevention and treatment of HF.

From the author’s visualized analysis, Yip H. K., Huang C. Y. and O. 'Rourke B. have more studies on the relationship between MF and HF, and their outputs are higher. Meanwhile, Abel E .D., Huang C. Y. and O. ′Rourke B. have higher H index, which indicates that they have made some achievements in this field. In recent years, the Abel E. D. from the University of Utah of the United States, focused mainly on the relationship between cardiac energy metabolism and HF (Lopaschuk et al., 2021). The latest results of the team show that pyruvate enters the mitochondria through the mitochondrial pyruvate carrier (MPC) for citrate cycle metabolism, and plays an important role in the regulation of myocardial metabolic function (Zhang et al., 2020c). Huang C. Y. Team from China Med Univ’s has been working on the treatment of HF through mitophagy for a long time, and has found that IGF-IIR can induce mitophagy through Rab9-dependent selective autophagy, which provides a new idea for the treatment of HF (Huang et al., 2018a; Huang et al., 2018b). The O. 'Rourke B. team has long been engaged in the research on the improvement of myocardial energy metabolism by regulating MFs in multiple ways. The team found that the inhibition of miR-181c expression can reduce the generation of reactive oxygen species (ROS) and reduce the calcium level of myocardial mitochondria, thus achieving the effect of preventing and controlling HF (Roman et al., 2020). From the analysis of the author’s cooperation in the figure, it can be seen that the Wang Wei team has more cooperation with other countries/regions, forming a larger cluster, while the Yip H. K. team and the Huang C. Y. team in Taiwan have no in-depth cooperation with other countries/regions.

This study also conducted a visualized analysis of the published journals. According to the H index, it can be seen that the articles on mitochondria and HF published by *Circulation* and *Circulation Research* have great influence. From the analysis of co-citation journals, most of the cited journals in this field are high-quality journals in cardiovascular medicine, molecular biology, biochemistry, life sciences and other fields. Uch as circulation, Journal of Biological Chemistry, Journal of Molecular and Cellular Cardiology, Nature, etc. According to the analysis of the top ten journals, studies on the relationship between mitochondria and HF have been published not only in the cardiovascular field, but also in the cellular and molecular fields, and most of these papers have been published in high-quality journals. This shows that the related basic research and clinical research have high academic value in the eyes of global scholars. It is suggested that MF research should be actively carried out in the future to promote its greater role in the prevention and treatment of HF and even cardiovascular field.

From the co-citation analysis, it was found that most of the cited literatures had high impact factors, and most of the studies focused on exploring the pathogenesis of HF from the perspectives of cardiac metabolic abnormalities, mitochondrial quality control and oxidative stress. It is well known that the onset of HF is accompanied by a series of changes in energy metabolism. Including the conversion of energy substrates, mitochondrial dysfunction and the depletion of high-energy phosphates, myocardial substrate metabolism in HF is severely impaired, and the selection and utilization of substrates such as fatty acids and glucose are reconstructed, which can further lead to the progress and deterioration of HF (Bertero and Maack, 2018a). Mitochondrial quality control refers to the continuous biogenesis, fusion, fission and autophagy of mitochondria, which is essential for heart health (Tahrir et al., 2019).

### 4.2 Hot spots and trends

Keyword analysis reflects the core and research focus of a document, while the in-depth analysis of keyword co-occurrence can identify the research hotspots and trends more quickly. In this part, the visualized clustering analysis of keywords, combined with the color clustering map and the emergence map, was used to analyze the hot spots and frontiers of the current research on mitochondria and HF. According to the results, oxidative stress, metabolic disorders, mitochondrial Ca^2+^homeostasis imbalance, mitochondrial quality control and inflammation-mediated mitochondrial dysfunction in the pathogenesis of HF are the research hotspots, which are summarized as follows.

### 4.3 Activation mechanism of mitochondrial dysfunction in HF

#### 4.3.1 Oxidative stress

Studies have confirmed that the damage caused by mitochondrial ROS (mROS) is the main pathogenic factor of HF. Scholars in early time generally believed that ROS is a by-product of mitochondrial respiration. With the deepening of research, people realized that when the generation rate of ROS exceeds the clearance rate or the endogenous antioxidant capacity is weakened, oxidative stress will occur, which will lead to heart damage (Wong et al., 2017; van der Pol et al., 2019). Excessive release of ROS in pathological conditions can induce nonspecific damage to cell membranes, proteins, and DNA, and these responses are particularly evident in mitochondria, where DNA and enzymes in the tricarboxylic acid (TCA) cycle are highly sensitive to oxidative damage. On the one hand, ROS-induced mitochondrial damage may in turn affect energy production. On the other hand, increasingly severe mitochondrial dysfunction can trigger sterile inflammation and cardiomyocyte death in the heart, which also contributes to the pathological progression of HF (Okonko and Shah, 2015). A research proposed that the elevated mROS is a major source of oxidative stress in HF and the reduced mROS *in vivo* may mitigate sudden cardiac death (SCD). It has been found that excess mROS may lead to HF and SCD by inhibiting or altering metabolic phosphorylation, antioxidants, and ion transporter networks. Reducing the level of mROS can prevent and reverse SCD and HF. At the same time, the results of this study also confirm the feasibility of mitochondria as potential therapeutic targets for HF and SCD (Dey et al., 2018). It has also been found that oxidative stress may interact with mitochondrial dysfunction in the development of HF. In the failing heart, ATP synthase activity is inhibited due to increased protein acetylation, which hinders ATP production, impairs electron flow and enhances ROS levels. The overproduced ROS interacts with cellular and mitochondrial components, such as proteins, DNA, lipids, and other molecules, which in turn exacerbate mitochondrial damage leading to more severe HF (Zorov et al., 2014). In addition, mROS has also been shown to be a molecular mediator of hypoxia signaling pathway, MAP kinase pathway, inflammatory pathway and retrograde communication between mitochondria and nucleus, but whether these mechanisms are related to the pathogenesis of HF still needs further research to prove (Hamanaka and Chandel, 2010; Ray et al., 2012; Javadov et al., 2014).

#### 4.3.2 Mitochondrial metabolic pathway

Normally, the energy metabolism of the heart is closely coordinated with the utilization of substrates to meet the energy needs of the human body. The oxidation of fatty acids, glucose, ketone bodies and other amino acids in myocardial mitochondria provides a large amount of ATP for the heart (Bedi et al., 2016). However, myocardial substrate metabolism is severely impaired in HF, and the selective utilization of substrates such as FA, glucose and ketone bodies is reconstructed, resulting in myocardial energy metabolism disorders, which further leads to the deterioration of HF (Bertero and Maack, 2018a). Down-regulation of fatty acid oxidation and increased glucose utilization in cardiac hypertrophy have been identified as markers of metabolic remodeling (Zhou and Tian, 2018). FA has always been regarded as the main “fuel” for heart operation, which can provide 70% of ATP for the heart (Bedi et al., 2016). It has been found that FA oxidation is significantly reduced in different HF models, so various FA oxidation stimulation schemes have been widely involved in the prevention and treatment of HF and have achieved some results (Bedi et al., 2016; Legchenko et al., 2018). However, it has also been found that increased FA uptake and oxidation rate may lead to the accumulation of lipotoxic metabolites and cardiac damage (Goldberg et al., 2012). Therefore, it still needs further exploration to find a reasonable treatment plan. Additionally, appropriate regulation of glucose metabolism may also be a potential treatment for HF. The dependence of the heart on glucose in pathological conditions leads to increased glycolysis. The decoupling of glycolysis from oxidation leads to increased cytoplasmic lactate production in mitochondria, which further reduces the efficiency of ATP synthesis. And exacerbates cardiac pathological remodeling (Bertero and Maack, 2018b). For example, sodium-glucose cotransporter 2 (SGLT2) inhibitors are effective hypoglycemic drugs, and some studies have found that SGLT2 inhibitors significantly reduce the risk of death and rehospitalization in patients with HF, but the specific mechanism still needs to be studied (Zannad et al., 2020). In recent years, more and more attention has been paid to the study of ketone body metabolism and HF. Some studies have found that the level of ketone body oxidation increases during HF, which is related to the increase of circulating ketone levels in patients with HF (Takahara et al., 2022). Another hypothesis is that ketones are more likely to function as signaling molecules in disease (Newman and Verdin, 2014). Although new research suggests that the increase in ketone body oxidation is cardioprotective (Monzo et al., 2021). However, it is still controversial whether increasing ketone body content or its oxidation can promote ATP production to further improve cardiac function.

#### 4.3.3 Dysregulated mitochondrial Ca^2+^homeostasis

Ca^2+^homeostasis imbalance has long been considered as a marker of HF (Santulli et al., 2015). It is well known that Ca^2+^ plays a central role in the regulation of excitation-contraction coupling (ECC) in cardiomyocytes. Under normal conditions, the transient increase of Ca^2+^ level in cardiomyocytes stimulates myocardial contraction. Mitochondria accumulate Ca^2+^ during contraction and participate in mitochondrial oxidative phosphorylation and ATP synthesis in the electron transport chain (ETC). In the early stages of HF, the increased energy demand of the heart triggers a complementary regulation of mitochondrial oxidative phosphorylation *via* Ca^2+^ and ADP, which ultimately leads to a disruption of Ca^2+^homeostasis in myocardial mitochondria. As a ubiquitous second messenger, Ca^2+^ is involved in the regulation of a variety of biological processes, including oxidative phosphorylation, ATP synthesis, ROS generation, mPTP opening and mitochondrial autophagy. These effects make Ca^2+^ an important regulator of MF (Xu et al., 2020). In addition, in the presence of mitochondrial Ca^2+^homeostasis imbalance, Ca^2+^ reuptake by the sarcoplasmic reticulum (SR) is impaired and Ca (TS4) leakage through ryanodine receptors (RYR) is increased, which results in a transient decrease in cytosolic Ca (TS4) during excitation but an increase in cytosolic Ca^2+^ at baseline, causing Ca^2+^ overload (Odagiri et al., 2009; Santulli et al., 2015). Ca^2+^ overload can lead to the opening of mitochondrial permeability transition pore (mPTP), the increase of mitochondrial oxidative stress, the collapse of mitochondrial membrane potential, the disorder of ATP production and the necrosis of myocardial cells, and finally induce HF (Cao et al., 2019; Smith and Eisner, 2019; Xu et al., 2020). The mechanism of Ca^2+^ overload is still unclear. The mitochondrial calcium uniporter (MCU) complex is an ion channel located in the mitochondrial inner membrane (IMM), which can transport Ca^2+^ into the mitochondrial matrix. It is essential to prevent mitochondrial Ca^2+^ overload and maintain mitochondrial Ca^2+^homeostasis (Yu et al., 2018). It has been found that the level of mitochondrial MCU protein in the heart of mice with pressure-induced cardiac hypertrophy is significantly increased, which implies that the level of MCU will increase compensatively when the myocardium is short of energy, resulting in increased mitochondrial Ca^2+^ uptake to promote ATP synthesis (Zaglia et al., 2017). However, it has also been found that MCU may mediate acute mitochondrial Ca^2+^ overload during acute ischemic injury, which ultimately induces cardiomyocyte death. Taken together, these finding provide insight into that potential of MCU complexes for target therapy of HF (Garbincius et al., 2020). On the other hand, the mitochondrial (Na^+^/Ca^2+^)exchanger (NCLX) is believed to be the main pathway for Ca^2+^ efflux to the cytoplasm, which is essential for maintaining Ca^2+^ homeostasis (Takeuchi and Matsuoka, 2021). Conditional knockout of NCLX has been found to lead to rapid fatal HF (Kostic and Sekler, 2019). Overexpression of NCLX has also been shown to increase mitochondrial calcium efflux and prevent the onset of HF by reducing the production of reactive oxygen species and limiting myocardial fibrosis in animal experiments. NCLX has strong research potential as a target for HF intervention (Zaglia et al., 2017).

#### 4.3.4 Mitochondrial quality control

Mitochondrial quality control refers to the coordinated biogenesis, mitochondrial dynamics and autophagy of myocardial mitochondria that maintain their normal morphology, quantity and quality, so as to ensure the normal function of mitochondria. Failure of mitochondrial quality control leads to cardiomyocyte dysfunction and ultimately to HF (Qiu et al., 2019). Defects in mitochondrial biogenesis have been found to reduce myocardial ATP supply and trigger mitochondrial dysfunction, peroxisome proliferator-activated receptor-γ coactivator-1α (PGC-1α) and SIRT1 and SIRT3 among type III histone deacetylases (sirtuins, SIRTs) have important effects on mitochondrial biogenesis. SIRT1 is the upstream regulator of PGC-1α, and overexpression of SIRT1 can enhance the deacetylation of PGC-1α and promote mitochondrial biogenesis. PGC-1α promotes the binding of the transcription factor estrogen related receptor *α* (ERRα) to the ERRα response element located in the SIRT3 promoter, which in turn activates SIRT3 expression (Koentges et al., 2015; Wang et al., 2020). Recent studies have also found that melatonin promotes mitochondrial biogenesis by increasing the expression of SIRT1 and PGC-1α. (Niu et al., 2020); The concept of mitochondrial dynamics was first proposed by Lewis et al., In 1914. The team believed that mitochondria changed their shape too frequently by fusion and fission to maintain the stability of their network structure (Lewis and Lewis, 1914). Mitochondrial fusion and fission occur mainly in the inner and outer mitochondrial membranes and are controlled by a group of dynamic-related regulatory proteins containing conserved GTPase domains. Mitochondrial fission occurs only in the outer mitochondrial membrane, and its regulatory proteins include dynamic-related protein 1 (Drp1), mitochondrial fission protein 1 (Fis1), and mitochondrial fission factor (MFF) (Westermann, 2010; Schmitt et al., 2018; Qiu et al., 2019). It has been found that activation of Drp1 can induce mitochondrial damage and cardiac dysfunction, while inhibition of Drp1 signaling can alleviate mitochondrial dysfunction during HF (Xia et al., 2017). Mitochondrial fusion is a beneficial process in which healthy mitochondria can replace damaged mitochondria and restore their activity to maintain normal metabolism. Mitochondrial fusion plays an important role in mitotic protein one and 2 (Mfn1, Mfn2), fuzzy onions 1 (Fzo1) and optic atrophy associated protein 1 (OPA1) (Gao and Hu, 2021). It has been found that disruption of mitochondrial fusion induced by Mfn1 and Mfn2 knockout can cause fatal HF (Chen et al., 2011). It has also been shown that OPA1-mediated mitochondrial fragmentation triggers dilated cardiomyopathy and HF in mice (Wai et al., 2015); Mitochondrial autophagy is an important mediator of mitochondrial quality control in cardiomyocytes. Under pathological conditions, cells achieve self-renewal and normal metabolism by activating autophagy and other pathways. In the process of mitophagy, defective mitochondria are labeled by ubiquitin chains, and then phagocytized into double-layer vesicles and autophagosomes, which are finally transmitted to lysosomes for degradation and clearance (Shirakabe et al., 2016). At present, mitochondrial autophagy in cardiomyocytes has attracted much attention, and PTEN-induced putative kinase-1 (PINK1) and E3 ubiquitin ligase Parkin-mediated autophagy are the most studied (Rub et al., 2017). The role of BAG3 in the regulation of mitochondrial dynamics and mitochondrial quality control in cardiomyocytes has also been reported, but the exact underlying mechanism remains unclear (Tahrir et al., 2017).

#### 4.3.5 Inflammation correlation with mitochondrial dysfunction

HF with reduced ejection fraction is associated with increased levels of proinflammatory cytokines, which reveals the potential role of the immune system in the pathogenesis of HF. However, the results of all treatments for HF inflammation in clinical trials so far are not satisfactory, and the pathogenic mechanism of inflammation in disease progression is still unclear (Adamo et al., 2021). Most HFs are aseptic inflammation, a chronic inflammatory state characterized by activation of the innate immune system. The innate immune system is activated by the recognition of specific receptors, namely, pattern recognition receptors (PRRs), by pathogen-associated molecular patterns (PAMPs) or damage-associated molecular patterns (DAMPs), which are derived from host cells and released in response to cellular stress or tissue injury. The NLRP3 inflammasome is an important part of the PPRs signaling pathway (Katsuumi et al., 2016). Recently, more scholars have found that NLRP3 inflammasome is closely related to the activation of mitochondrial DAMPs, and the activation of NLRP3 inflammasome is one of the important mechanisms to ensure the normal function of mitochondria (Zhang et al., 2010; Dela and Kang, 2018). Currently, it has been found that overexpression of NOX1 and NOX4 in cardiomyocytes can activate dynamic-related protein 1 (Drp1) and induce mitochondrial fission, which further activates NLRP3-mediated cardiomyocyte apoptosis in a caspase-1-dependent manner, resulting in myocardial dysfunction (Zeng et al., 2020).

### 4.4 Research progress of mitochondrial targeting therapy for HF

At present, the treatment of HF has entered a stage that exploring the pathogenesis of HF and finding the corresponding treatment are urgent. In the past, the drugs for the treatment of HF mainly included beta blockers, angiotensin-converting enzyme inhibitors, angiotensin receptor blockers (ARB), vasodilators and diuretics. It works mainly by reducing the load on the failing heart (Brown et al., 2017). Based on the above analysis, the recovery of MF in the treatment of HF is the best way to fundamentally solve the problem of energy imbalance, and mitochondria can be used as an important target for the development of therapeutic intervention drugs for HF. Several drugs targeting treating HF have been listed, mainly through the prevention of oxidative damage, regulating myocardial substrate metabolism, improving mitochondrial quality control, maintaining mitochondrial Ca^2+^homeostasis, and anti-inflammatory.

Coenzyme Q10 (CoQ10) is a fat-soluble quinone present in all cells. It is one of the substances involved in the electron transport chain and aerobic respiration in the mitochondria of eukaryotic cells. As a natural antioxidant, CoQ10 has been shown to be effective in a variety of metabolic diseases (Martelli et al., 2020). At present, the role of CoQ10 in the treatment of HF has been generally recognized. In 1994, an Italian study of 2664 patients with HF for 3 months found that CoQ10 could significantly improve the clinical sign and symptom of patients, improve the quality of life of patients, and has a high safety (Baggio et al., 1994). MitoQ is a mitochondria-targeted antioxidant with strong antioxidant activity (Botting et al., 2020). MitoQ has been shown to improve mitochondrial dysfunction in rat HF by reducing hydrogen peroxide formation, improving mitochondrial respiration, and modulating mPTP opening (Ribeiro et al., 2018). Randomized controlled studies have also shown that oral administration of MitoQ for 6 weeks can improve vascular endothelial function and reduce aortic stiffness in the elderly, but there are few clinical trials on the specific treatment of HF (Rossman et al., 2018); Studies have shown that SGLT2 inhibitor can benefit patients with cardiovascular disease by improve substrate availability and myocardial energy metabolism. Commonly used SGLT2 inhibitors are canagliflozin, dapagliflozin, empagliflozin and ertugliflozin. They decrease the maximal ability of the renal tubules to reabsorb glucose, thereby increasing the excretion of glucose in the urine and decreasing the concentration of plasma glucose, thereby improving glucose metabolism (Cowie and Fisher, 2020). It has also been found that trimetazidine can improve isoproterenol-induced myocardial metabolic remodeling in HF rats by activating AMPK and PPARα to regulate myocardial substrate utilization, especially ketone body metabolism (Li et al., 2020); It has been found that mitochondrial quality control regulators are also potential new drug targets in mitochondrial therapy. Mdivi-1 is an inhibitor of mitochondrial fission protein Drp1, and early studies have shown that it may improve the therapeutic effect of myocardial infarction by altering mitochondrial dynamics. However, the use of mdivi-1 treatment in clinically relevant large animal models of myocardial infarction did not reduce the size of myocardial infarction or improve cardiac function, so the relevant studies still need to be validated in large samples (Ong et al., 2019); CDGSH iron-sulfur domain protein 2 (Cisd2) is critical for mitochondrial integrity and intracellular Ca^2+^homeostasis. Cisd2 deficiency disrupts Ca^2+^homeostasis by inducing dysregulation of muscle/endoplasmic reticulum Ca^2+^-ATPase (Serca2a) activity and leads to mitochondrial Ca^2+^overload in cardiomyocytes. It has been found that Cisd2 activators can up-regulate Cisd2 expression, enhance mitochondrial and Serca2a activity, and maintain intracellular Ca^2+^homeostasis. Thereby reducing age-related cardiac dysfunction and slowing the progression of HF. In addition, the study highlights Cisd2 as a novel drug target for the development of therapies to delay cardiac dysfunction (Yeh et al., 2019); The relationship between inflammation and the onset of HF has long been recognized, but current therapies aimed at reducing inflammation in patients with HF have not shown appreciable results (Adamo et al., 2020). The hypothesis that the interleukin-1β inhibitor canakinumab reduces the hospitalization rate and mortality rate in patients with HF has been proposed, and the final results show that the efficacy of canakinumab increases in a dose-dependent manner, which is convincing (Everett et al., 2019). In conclusion, the development of new treatments for mitochondrial dysfunction has great research potential, which is of great practical significance to reduce the economic burden of patients with HF and improve their symptoms.

## 5 Limitations

This study is based on the WoSCC database for visualized analysis, and some limitations are: (van der Meer et al., 2019): The retrieval time of this study is limited to the literature published in the past 20 years from January 2002 to December 2021, but with the update of the WoSCC database, the actual number of literature will continue to change; (Ziaeian and Fonarow, 2016); Because the bibliometrics software can not distinguish the author’s initials at present, there may be wrong conclusions in the author’s analysis part of this paper; (Yancy et al., 2017); Due to the limited number of keywords included in the literature, some core words may be omitted in the analysis process, so the analysis results may be affected; (Yancy et al., 2013); Because the retrieval of this study was limited to the SCI-E index database in WOS, the literature not included in the SCI-E index database was not included in the analysis. However, the results of this study reveal the research status, hot spots and trends of the relationship between MF and the pathogenesis of HF, which can provide some reference for researchers to further explore in this field.

## 6 Conclusion

Based on the WoSCC database, this study conducted a bibliometric analysis of 4236 studies published from 2002 to 2021 on the relationship between MF and HF. The results show that the number of papers on the relationship between mitochondria and HF has increased year by year in the past 20 years, and China and the United States are the leading countries in this field. At the same time, UNIVERSITY OF CALIFORNIA SYSTEM is the research institution that has the greatest impacts on the research results, and Yip H K is the most fruitful author in this field. American Journal of Physiology-heart and Circulatory Physiology is probably the most popular journal, and most of the articles published on mitochondria and HF are cited from internationally influential journals. In addition, oxidative stress, metabolic dysfunction, mitochondrial Ca^2+^homeostasis imbalance, mitochondrial quality control and mitochondrial dysfunction mediated by inflammation in the pathogenesis of HF are the hot spots. To sum up, the study of the relationship between mitochondria and HF has broad prospects for development, and targeted regulating of mitochondria will also be the focus of future research on the prevention and treatment of HF.

## Data Availability

The raw data supporting the conclusion of this article will be made available by the authors, without undue reservation.
